# The Impact of Micronutrients-Calcium, Vitamin D, Selenium, Zinc in Cardiovascular Health: A Mini Review

**DOI:** 10.3389/fphys.2021.742425

**Published:** 2021-09-09

**Authors:** Harini Narayanam, Suresh V. Chinni, Sumitha Samuggam

**Affiliations:** ^1^Department of Physiology, Manipal University College Malaysia (MUCM), Melaka, Malaysia; ^2^Department of Biotechnology, Faculty of Applied Sciences, AIMST University, Bedong, Malaysia

**Keywords:** cardiac health, micronutrient, selenium, zinc, calcium, vitamin D

## Abstract

The role of micronutrients in health and disease has increased the curiosity and interest among researchers. The prime focus of this review is the significance of trace elements- calcium, vitamin D, selenium and zinc with cardiovascular health. WHO identified cardiovascular diseases (CVD) as the leading cause of deaths globally. Identifying the risk factors that could be modified and creating new treatment strategies remains to be the main concern for CVD prevention. The data that showed the relationship between trace elements and various ways in which they may contribute to cardiovascular health and disease from clinical trials and observational studies were collected from databases such as PubMed and Embase. Based on these collected data, it shows that either high or low circulating serum levels can be associated with the development of cardiovascular diseases. Micronutrients through diet contribute to improved cardiac health. However, due to our current lifestyle, there is a huge dependency on dietary supplements. Based on the observational studies, it is evident that supplements cause sudden increase in the circulating levels of the nutrients and result in cardiovascular damage. Thus, it is advisable to restrict the use of supplements, owing to the potent risks it may cause. In order to understand the exact mechanism between micronutrients and cardiac health, more clinical studies are required.

## Introduction

**FIGURE 1:**
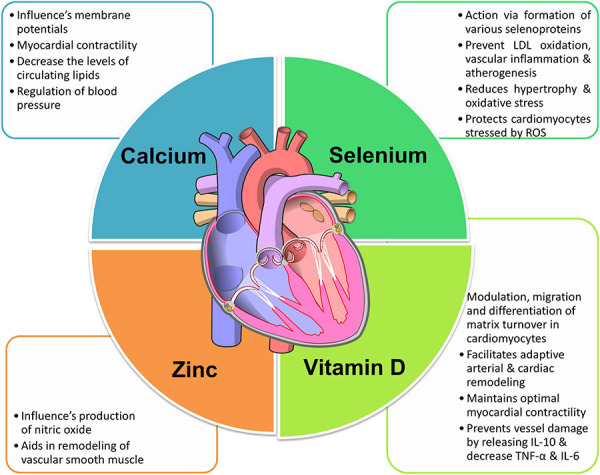
The role of micronutrients in cardiovascular health. Image is adapted from https://commons.wikimedia.org/wiki/File:Diagram_of_the_human_heart_(no_labels).svg (Stenemo, 2017).

A staggering 31% of mortality worldwide can be primarily due to cardiovascular disease. Nearly 85% of these deaths are due to heart attacks and strokes. A combination of risk factors like unhealthy diet and obesity, physical inactivity, hyperlipidemia, stress, and hereditary factors contribute to developing cardiovascular diseases (CVD) (Roth et al., 2017; World Health Organization, 2021). Identifying the risk factors that can be modified and treatments remains to be a main concern for CVD prevention. The pivotal role micronutrients and trace elements play in health and disease has paved the way for numerous scientific research. Additionally, studies on how supplementation might improve health status have gained immense popularity (Gać et al., 2021). There seems to be an uncertainty in deciding if the supplements confer overall health or if they work only in parts. It cannot be said that all the health supplements are 100% safe to use. The current review analyses the importance and effects of some micronutrients and trace elements and their supplements on cardiovascular health as illustrated in Figure 1.

### Biological Role of Calcium in Bone Health and Cardiovascular System

Calcium plays an important role in the biological system but is a pivotal mineral in bone health and cardiovascular system (Cano et al., 2018). The daily dietary intake of calcium for both men and women is 1,200–1,500 mg. Milk and milk products, green leafy vegetables that include kale, spinach etc. and fortified flour are the major sources of calcium. To achieve the adequate serum levels through diet alone is quite challenging which is why many people opt for calcium supplements independently or in conjunction with vitamin D to achieve the adequate serum levels and enhance bone health (Michos et al., 2021). There have been mixed results on the role of calcium and its supplements in cardiovascular health based on numerous observational and clinical trial studies over the time. Concentration of extracellular calcium directly influences cell membrane potentials of all excitable tissues especially heart and nerves. Calcium is important for muscle contraction, including the myocardium (Elsevier, 2021). In a study conducted on patients admitted for acute coronary syndrome, it was observed that hypocalcaemia was independently correlated with poor long term cardiovascular outcomes (Wang et al., 2010). Similarly, Schmitz et al. (2021) have also postulated in their study that decreased serum calcium levels were independently linked with increased mortality after acute myocardial infarction, but no proof is available to show that calcium supplementation would benefit. In some animal studies, excess calcium intake lowered blood cholesterol level of rats by inhibiting the absorption of fatty acids. Similarly studies on humans showed a better regulation of blood pressure, decreased lipid levels and improved glycemic control all of which protect against development of CVD (Rautiainen et al., 2013). Calcium supplements proved beneficial and reduced mortality rate in a cohort study done on post-menopausal women (Shin et al., 2012). Lewis et al. (2015) in a meta-analysis study documented that increased calcium supplements did not significantly augment the risks for myocardial infarction, angina pectoris and acute coronary syndrome in elderly women. In their meta-analysis Wang et al. (2021) have reported that calcium intake either lesser or more than 800 mg per day was related with increasing risk of cardiovascular mortality and they did not find any evidence to associate calcium supplements with cardiovascular mortality. Mursu et al. (2011) and Prentice et al. (2013) in their cohort studies on women have reported that participants taking calcium exhibited lowered risk of developing cardiac diseases. Wang et al. (2021) reported that calcium supplements were not responsible for vascular calcification in either men or women.

On the contrary, increase in the levels of serum calcium is correlated with a potential risk of CVD like hypertension, myocardial infarction (MI) etc. and there are several meta-analysis reports in support (Kwak et al., 2014; Reid et al., 2016). Studies have shown that hypercalcemia promotes vascular calcification including coronary artery and aortic calcification and increases the formation of coronary artery plaque (Shin et al., 2012; Kwak et al., 2014). An analysis of 2,742 atherosclerotic adults revealed that increasing the dietary consumption of calcium decreased the risk of incident coronary atherosclerosis whereas substituting with calcium supplements increased the risk by 22% (Anderson et al., 2016). Hypercalcemia and hypercalciuria was reported by Gallagher et al. (2014) in participants who were taking the recommended calcium intake of 1,200 mg/day. In a meta-analysis of observational studies Chung et al. (2016) indicated that there is no association of dietary calcium on development of cardiovascular diseases including strokes and coronary heart disease (CAD) while supplement of calcium may surge the risk of coronary heart disease especially myocardial infarction. Lewis et al. (2015) in their meta-analysis of randomized controlled trials described that supplementing calcium either independently or along with vitamin D increased the incidence of coronary heart disease in older women. Larsson et al. (2017) have reported that a higher serum calcium level led to genetic susceptibility and was correlated with the increased threat of CAD and myocardial infarction. Calcium supplements cause a surge in the ionized and total serum calcium levels in a short interval of time after ingestion and this increases the levels of insoluble calciprotein particles which are calcification activators in the serum that may lead to vascular calcification and myocardial infarction. Whereas calcium coming from dietary sources results in a slow and controlled increase in calcium levels (National Institutes of Health, 2011).

Since there are studies reporting the downside of increased calcium levels, especially on cardiovascular health, there is some concern regarding calcium supplements. In view of the increasing concerns, it would be advisable to rely on the dietary sources of calcium to meet the daily recommended levels and limit the usage of supplements.

### Correlation of Low Level of Vitamin D With the Risk of Cardiovascular Diseases

Cholecalciferol or vitamin D3 is a steroid hormone naturally available, and it can also be obtained from consumption of seafood especially salmon, tuna and eel, cow milk, and eggs which are rich sources of vitamin D. Vitamin D is absorbed in the small intestine. The skin naturally synthesizes cholecalciferol from 7-dehydrocholesterol when exposed to ultraviolet B rays (UVB). Cholecalciferol is hydroxylated to 25-hydroxyvitamin D [25(OH)D], the major metabolite that circulates in blood. It is transformed into 125-dihydroxyvitamin D (calcitriol) the active form in the kidneys by the 1α-hydroxylase enzyme.

Deficiency of vitamin D is a global problem and some of the causes included is diminishing synthesis in the skin due to aging, hyperpigmentation, low dietary intake, limited exposure to sun, pollution, smoking, obesity (Norman and Powell, 2014). The daily recommended dietary allowance of vitamin D for individuals aged between 1 and 70 years is 600 IU and for adults older than 70 years it is 800 IU/day. This value accounts to a serum 25-hydroxyvitamin D level of 20 ng/mL or greater based on bone health (National Institutes of Health, 2011).

Lower levels of vitamin D in the blood has been linked with higher cardiovascular risk in numerous observational studies, epidemiological investigations, and laboratory studies (Al Mheid and Quyyumi, 2017). Several cells in the vascular system have the receptors for vitamin D. The enzyme 1α-hydroxylase, that converts 25-hydroxyvitamin D to calcitriol is produced in the vascular smooth muscle, endothelial cells, and cardiomyocytes (Danik and Manson, 2012). Calcitriol modulates proliferation, migration and differentiation and matrix turnover in the cardiomyocytes, modulates the osteoclastic gene expression in smooth muscle and thereby facilitates adaptive arterial and cardiac remodeling. It maintains optimal endothelial function and vascular tone, regulates calcium flux and sarcomere function in the cardiomyocytes to confer optimal myocardial contractility (Norman and Powell, 2014). Some studies have shown that vitamin D by its influence on PTH, indirectly on calcium metabolism has an important role in vascular calcification. Both hypo and hypervitaminosis of vitamin D can cause calcification of blood vessels (Zebger-Gong et al., 2011).

Low levels of vitamin D probably increase CVD risk by altering pre-determined cardiovascular risk factors like hypertension, diabetes, and inflammation (Chin et al., 2017). There were many observational studies that found a reverse relationship between blood pressure and serum vitamin D levels (Burgaz et al., 2011) but the findings were not very significant (Danik and Manson, 2012). The possible mechanism may involve diminishing the proliferative effects of renin-angiotensin-aldosterone system on vascular smooth muscle cells (Ajabshir et al., 2014). Calcitriol by binding to the promoter region in the gene *REN-1C* represses renin expression, and aids in lowering the probability of emerging hypertension. In addition, the renal arteries of individuals with diminished levels of vitamin D have low expressing angiotensin-l receptors (Mursu et al., 2011). In addition, evidence from some animal studies had related the direct effect of vitamin D on endothelium and vasculature and how it protects against endothelial dysfunction (Lubos et al., 2010).

Effect of vitamin D on parathyroid hormone (PTH) and calcium metabolism is also suggested as one of the mechanisms which alter the blood pressure. Minimal levels of vitamin D will increase PTH which in turn constricts the vasculature and leads to hypertension by increasing intracellular calcium levels (Fujii, 2018). Supplementation of vitamin D has been proven beneficial in treating high blood pressure in some ethnic and age groups as observed in some case-control studies (Judd et al., 2010) but it did not show any significant effect on non-hypertensive population. Based on this supposition the consumption of vitamin D supplements as an antihypertensive agent must not be advocated (Wang et al., 2010).

Many epidemiological studies have correlated lower serum vitamin D levels to increased incidence of type 2 diabetes mellitus. Diminished levels of vitamin D are assumed to increase insulin resistance, cause damage to the beta-cells of the pancreas, and consequently decrease secretion of insulin (Chin et al., 2017). There is conflicting data from various clinical studies and the valuable influence of vitamin D on treating or preventing diabetes cannot be ascertained due to lack of supporting evidence. Several systematic reviews and meta-analyses also established that HbA1c levels were not lowered even after supplementing vitamin D and there was no improvement in the function of pancreatic beta-cells and insulin resistance (Chin et al., 2017).

Vitamin D has shown some protective influence on vessel walls as observed in some research papers. Vessel walls are shielded against damage from inflammation by enhancing the expression of anti-inflammatory cytokines like IL-10 and by reducing the expression of pro-inflammatory proteins like, TNF-α and IL-6 (Zittermann et al., 2005). But there is not sufficient evidence to corroborate the findings.

Although vitamin D is dynamically involved in several pathways in the homeostasis of the cardiovascular health and low vitamin D levels are recognized as a menace for chief cardiovascular diseases including hypertension, atherosclerosis, strokes, heart failure etc. the supporting evidence available is limited. It is essential to prolong studying the outcomes of vitamin D on cardiovascular health with more emphasis on the pathology before advocating the use of vitamin D supplements for improving cardiovascular health to the public.

### Trace Element Zinc in Development of Cardiovascular Diseases

Zinc has numerous functions in the human body and therefore is recognized as an important trace element. It is a vital antioxidant that prevents the formation and reactive response of free radicals which may damage the cells and lead to degenerative diseases (Chabosseau and Rutter, 2016). Daily allowance of zinc for men and women is 11 and 8 mg, respectively, and should never exceed a maximum amount of 40 mg/day (Ruz et al., 2019). The main dietary sources of zinc are meat that include beef, veal, pork, lamb, cereals, grains, fish, vegetables, nuts, milk, and dairy products (Olza et al., 2017). Zinc by being an important part in the metabolism of nucleic acids and protein biosynthesis warrants the proper course of cell growth, division, and functioning (Plum et al., 2010).

Deficiency of zinc is correlated with the development of cardiovascular diseases, mainly atherosclerosis (Chiu et al., 2004). Impaired function of superoxide dismutase causes oxidative stress which in turn increases the turnover of nitric oxide (NO) a potent vasodilator which can alter blood pressure. Also, increased oxidative stress can lead to atherosclerosis. A study conducted on the mice showed that when zinc intake was insufficient, there is increase in concentration of lipoproteins, augmented refashioning of vascular smooth muscle, augmented inflammation, and induced atherosclerotic plaque formation (Chiu et al., 2004). Zinc helps in maintaining adequate functioning of superoxide dismutase and keeps a check on NO generation (Ruz et al., 2019). However, studies on humans are still sparse and sufficient evidence is lacking to corroborate the findings.

Role of zinc in regulating blood pressure may be because of its antioxidant property and its role in regulating the flow of calcium ions. In a study conducted on mice, it was seen that zinc deficient diet increased the blood pressure in mice. Reduced zinc levels lead to hypertension, hypercholesterolemia and a higher BMI in a study conducted on human subjects. The association was statistically significant (Braun et al., 2018). Possible mechanism could be that deficiency of zinc stimulates retention of sodium in distal renal tubules (Williams et al., 2019). There have been controversial studies which showed an increase in hypertension with a rise in zinc levels and some studies showed no correlation between hypertension with either increase or decrease in the serum zinc levels (Gać et al., 2021).

Oxidative stress can trigger cellular and molecular destruction and aids in the development of insulin resistance which will lead to type 2 diabetes mellitus, which is a predetermined risk factor of cardiovascular disorders (Bao et al., 2010). Zinc is responsible for the production and maturation of insulin (Chabosseau and Rutter, 2016). Zinc can stifle the secretion of cytokines IL-1b, TNF-a, and IL-6 s from macrophages and monocytes, which may cause apoptosis of the pancreatic beta cells (Ruz et al., 2019). In a meta-analysis study, it was observed that zinc when supplemented at a low dose over prolonged period helped fasting blood glucose, improved insulin resistance, reduced triglycerides, total cholesterol, and LDL cholesterol (Pompano and Boy, 2021). But in another study women who ingested additional zinc doubled up their risk of developing cardiovascular occurrences (Gać et al., 2021). Although statistically significant results were seen with zinc supplementation on improving the blood cholesterol picture and on blood sugar levels nothing positively significant was reported on benefits of cardiovascular health (Khazdouz et al., 2020). Furthermore, some studies have reported a clear association between greater intake of zinc and the upsurge in total and cancer-related mortality (Shi et al., 2018).

Although there is some evidence showing the role and benefits of zinc in cardiovascular health, it is not enough to rationalize its supplementation. More research must be done, and more steadfast and consistent experimental data would be desired to arrive at a decision.

### Selenium in Cardiovascular Health

Selenium is another important trace element which influences cardiovascular health. It has a vital role in the formation of selenoproteins, so far about 30 of them are identified (Tinggi, 2008). The recent interest in selenium is integrated into important amino acid derivatives, such as selenomethionine, selenocysteine, methylselenocysteine, and selenocystathionine. Selenoproteins have important role in the body some of which include reducing hydrogen peroxide and organic peroxides, control cellular proliferation and apoptosis, regulate thyroid hormone levels, adjusts the work of cardiovascular system (Gać et al., 2021). The recommended dietary allowance for selenium is 55 μg/day to meet the nutritional needs of all healthy adults. Plant foods, meat, cereals, and seafood are the major dietary sources of selenium (Flores-Mateo et al., 2006).

Deficiency of selenium has been associated with CVD from a long time and an example is the Keshan disease a type of cardiomyopathy seen in some parts of China that has soil low in selenium (Alexander et al., 2020). The underlying mechanism that explains how selenium deficiency causes deterioration of cardiomyocytes and increases the predisposition to damage is yet to be discovered (Sun et al., 2019). There are several other studies that have conveyed an association between decreased selenium and increased danger of MI (Suadicani et al., 1992), acute coronary syndrome (Lubos et al., 2010). A meta-analysis on pooled data indicated that a body with physiologically high selenium levels exhibited lessened risk of CVD and mortality when compared to low body selenium status. Analogous results were seen in other meta-analysis reports by Flores-Mateo et al. (2006), Alexander et al. (2020), Zhang et al. (2016) etc. Regions that had very low selenium levels in the soil exhibited the protective effect of selenium and benefited from its supplementation (Kuria et al., 2020). Jenkins et al. (2020) in the meta-analysis described that supplementing selenium along with antioxidant mixture proved to be beneficial for CVD.

A meta- analysis report by Xiang et al. (2020) validated that circulating selenium levels do not influence the mortality rate of coronary diseases and the supplements do not help much (Witham et al., 2009). Vinceti et al. (2019) in their study have shown that increased exposure to selenium resulted in increased blood pressure. Selenium if taken in excess (900 μg/day) can cause digestive system disorders, hair loss, skin lesions, along with disturbances of endocrine, respiratory, and nervous systems (Gać et al., 2021). Sufficient data from clinical studies is not available to show the beneficial effects of selenium supplementation in cardiovascular disease treatment or prevention. There is a strong need of more evidence, before prescribing supplements of selenium for cardiovascular health because the benefits of selenium supplementation are uncertain, and their arbitrary usage carries a probability of toxicity (Danik and Manson, 2012; Benstoem et al., 2015; Khazdouz et al., 2020).

Owing to the ill effects supplements have on the health of the individual, it is better to rely on naturally available sources. A summary of the daily requirement and dietary sources has been depicted in the Table 1.

**TABLE 1:** Recommended dietary allowance, normal serum concentration, dietary sources, and deficiency symptoms of calcium, Vitamin D, selenium, and zinc.

**Micronutrient**	**Recommended dietary allowance**	**Normal serum concentration**	**Dietary sources**	**Deficiency symptoms**
Calcium	1,200–1,500 mg/day	9–11 mg/dL	Milk, cheese, okra, spinach, kale, sardines, pilchards, breads made from fortified flour	Hypotension, heart failure
Vitamin D	15–20 μg/day	30–100 ng/mL	Salmon, sardines, herring, mackerel, red meat, liver, egg yolk, fat spreads, breakfast cereals	Atherosclerosis, hypertension
Selenium	55 μg/day	70–150 ng/mL	Brazil nuts, seafood, eggs, organ meat, red meat, grains, cereals	Atherosclerosis, hypertension, hypercholesterolemia
Zinc	8–11 mg/day	0.7–1.6 μg/mL	Mushrooms, spinach, broccoli, kale, garlic, chickpea, lentils, beans, pumpkin seeds, pine nuts	Myocardial infarction, acute coronary syndrome

## Conclusion

Based on the large observational studies and available data, we conclude that, there definitely is an important role of the micronutrient’s calcium, zinc, vitamin D, and selenium on cardiovascular health. However, high circulating levels of these micronutrients have a detrimental effect on the heart health as evidenced in few of the studies, although the underlying mechanism is not very clearly understood. Micronutrients taken in the form of supplements cause an immediate surge in the serum levels for a prolonged period and the increment is sustained for longer hours. Further clinical trials and studies are needed to establish the benefits of supplements and it is important to weigh the risks before taking them. It is, therefore, advisable to depend on dietary sources to meet the daily requirement of micronutrients.

## Author Contributions

HN and SC equally contributed in terms of conception of the work. HN performed database search, drafting of the manuscript. HN, SC, and SS edited and reviewed the manuscript. SC procured the grant for the publication. All the authors have given their consent for submission.

## Conflict of Interest

The authors declare that the research was conducted in the absence of any commercial or financial relationships that could be construed as a potential conflict of interest.

## Publisher’s Note

All claims expressed in this article are solely those of the authors and do not necessarily represent those of their affiliated organizations, or those of the publisher, the editors and the reviewers. Any product that may be evaluated in this article, or claim that may be made by its manufacturer, is not guaranteed or endorsed by the publisher.
